# Extracellular superoxide dismutase inhibits hepatocyte growth factor-mediated breast cancer-fibroblast interactions

**DOI:** 10.18632/oncotarget.22379

**Published:** 2017-11-10

**Authors:** Briana Ormsbee Golden, Brandon Griess, Shakeel Mir, Matthew Fitzgerald, Charlotte Kuperwasser, Frederick Domann, Melissa Teoh-Fitzgerald

**Affiliations:** ^1^ Department of Biochemistry and Molecular Biology, Fred and Pamela Buffett Cancer Center, College of Medicine, University of Nebraska Medical Center, Omaha, NE 68198, USA; ^2^ Department of Surgery-General Surgery, University of Nebraska Medical Center, Omaha, NE 68198, USA; ^3^ Developmental, Molecular and Chemical Biology, Tufts University School of Medicine, Boston, MA 02111, USA; ^4^ Free Radical and Radiation Biology Program, Radiation Oncology, University of Iowa, Iowa City, IA 52241, USA

**Keywords:** tumor-stroma interactions, EcSOD, breast cancer, c-Met, cancer-associated fibroblasts

## Abstract

We have previously shown tumor suppressive effects of extracellular superoxide dismutase, EcSOD in breast cancer cells. In this study, an RTK signaling array revealed an inhibitory effect of EcSOD on c-Met phosphorylation and its downstream kinase c-Abl in MDA-MB231 cells. Moreover, an extracellular protein array showed that thrombospondin 1 (TSP-1), a scavenger of the c-Met ligand, hepatocyte growth factor (HGF) is significantly up-regulated in EcSOD overexpressing cells (Ec.20). We further determined the effects of EcSOD on HGF/c-Met-mediated cancer-fibroblast interactions by co-culturing normal fibroblasts (RMF) or RMF which overexpresses HGF (RMF-HGF) with MDA-MB231 cells. We observed that while RMF-HGF significantly promoted Matrigel growth of MDA-MB231, overexpression of EcSOD inhibited the HGF-stimulated growth. Similarly, a SOD mimetic, MnTE-2-PyP, inhibited HGF-induced growth and invasion of MDA-MB231. In addition, a long-term heterotypic co-culture study not only showed that Ec.20 cells are resistant to RMF-HGF-induced invasive stimulation but RMF-HGF that were co-cultured with Ec.20 cells showed an attenuated phenotype, suggesting an oxidative-mediated reciprocal interaction between the two cell types. In addition, we demonstrated that RMF-HGF showed an up-regulation of an ROS-generating enzyme, NADPH oxidase 4 (Nox4). Targeting this pro-oxidant significantly suppressed the activated phenotype of RMF-HGF in a collagen contraction assay, suggesting that RMF-HGF contributes to the oxidative tumor microenvironment. We have further shown that scavenging ROS with EcSOD significantly inhibited RMF-HGF-stimulated orthotopic tumor growth of MDA-MB231. This study suggests the loss of EcSOD in breast cancer plays a pivotal role in promoting the HGF/c-Met-mediated cancer-fibroblast interactions.

## INTRODUCTION

One of the main features of cancer cells is a persistent oxidative stress or a higher steady state levels of reactive oxygen species (ROS), when compared to their normal counterparts. The roles played by ROS in the hallmarks of cancer have been well reviewed [1]. ROS such as superoxide anion (O_2_^•−^), hydroxyl (^•^OH), and hydrogen peroxide (H_2_O_2_) exert a multifaceted role at the cellular level. While high levels of ROS can induce bio-molecular damage and even cell death when the levels exceed the anti-oxidative capacity of cells to detoxify them, moderate levels of ROS are essential for physiological signaling in promoting proliferation and differentiation. In normal cells, redox homeostasis is tightly regulated by balancing ROS generation with their scavenging systems. However, in malignant cells aberrant regulation of redox homeostasis is often observed. Activation of oncogenes, aberrant metabolism, and defective tumor suppressor genes are intrinsic factors known to promote ROS production in cancer cells, partly through activation of ROS-generating enzymes such as NADPH oxidases [2]. The enhanced oxidative stress observed in cancer cells can result not only from ROS overproduction, but also from decreased levels or inactivation of antioxidants such as superoxide dismutases (SODs).

We have previously shown that expression levels of the extracellular form of SOD (EcSOD or SOD3) is significantly down-regulated in a majority of breast carcinomas and its expression levels inversely correlated with clinical stage [3]. Overexpression of this antioxidant in breast cancer cells inhibited *in vitro* proliferation, clonogenic survival, invasion, tumor growth and metastasis [3, 4]. EcSOD is the only extracellular enzyme that scavenges superoxide (O_2_^•−^) which is the essential first step in eliminating the downstream production of more potent ROS (e.g. H_2_O_2_, ^•^OH, and ONOO^−^). Considering its unique cell-surface localization and the fact that the substrate for EcSOD, O_2_^•−^, crosses membranes poorly, loss of EcSOD is anticipated to promote an oxidative extracellular environment that will likely alter autocrine and/or paracrine signal transductions initiated at cell surface receptors.

Receptor tyrosine kinases (RTKs) such as the epidermal growth factor receptor (EGFR) have been shown to be ROS-sensitive or utilize ROS-dependent mechanisms for activation [5]. ROS can activate signal transduction pathways by oxidizing and therefore inhibiting the cysteine regions of the active sites of protein tyrosine phosphatases (PTPs), which promotes activation of tyrosine kinases. Recently, oxidative stress has also been suggested to be involved in activating c-Met signaling [6]. Therefore, loss of EcSOD expression and the resulting increase in extracellular O_2_^•−^ is expected to promote HGF/c-Met signaling. Upon activation, c-Met undergoes phosphorylation that evokes a variety of oncogenic responses leading to increased proliferation, scattering and motility, invasion, survival, angiogenesis, and metastasis [7]. In breast cancer, c-Met is overexpressed in 20–30% of cases, and is a strong, independent predictor of decreased survival [8]. Moreover, several types of signal cooperation and cross-talk between c-Met and EGFR pathways have been demonstrated in recent years [9]. This has driven the development of inhibitors that target c-Met as an anti-cancer strategy [10].

In addition to overexpression, c-Met activation can be promoted through mutations, or autocrine signaling in malignant cells. Mutations in c-Met that confer its constitutive activation independent of its ligand (hepatocyte growth factor, HGF) has been observed and autocrine up-regulation of HGF has been reported in lung cancer [11]. However, breast cancer cells rarely express the c-Met ligand, but often acquire HGF from stroma fibroblasts or the cancer-associated fibroblasts (CAFs) via a paracrine interaction [12]. CAFs are now recognized to be one of the key players in the tumor microenvironment that not only supports cancer cells but also interacts with the other stroma cells such as macrophages and endothelial cells, in a community fashion, to promote cancer cell proliferation, survival, malignant progression, angiogenesis, and metastasis [13]. These fibroblasts differ from the normal quiescent fibroblasts in their activated phenotypes and their pro-tumorigenic secretory profile. Pro-inflammatory [14] and pro-oxidative [15] phenotype have also recently been associated with CAFs. Understanding factors that contribute to the paracrine HGF/c-Met signaling during cancer-fibroblast interactions will therefore have a positive clinical impact for c-Met overexpressing cancers.

The objective of this study is to determine the effect of EcSOD and oxidative stress on HGF/c-Met-signaling in breast cancer. We found that overexpression of EcSOD inhibited HGF-mediated c-Met phosphorylation and resulted in an inhibition of three-dimensional (3D) Matrigel growth of MDA-MB231. This antioxidant enzyme also inhibited HGF-mediated cancer-fibroblast interactions in our co-culture model. In a prolonged co-culture study where breast cancer cells were seeded for multiple passages with mammary fibroblasts, followed by re-isolation of individual cell types, overexpression of EcSOD significantly suppressed the invasiveness of breast cancer cells that were pre-exposed to HGF-secreting fibroblasts, when compared to the parental MDA-MB231 cells. Concurrently, the isolated HGF secreting fibroblasts, which were co-cultured with EcSOD–overexpressing MDA-MB231 cells showed attenuated phenotype in their ability to promote naïve breast cancer cell invasion.

Furthermore, we have shown that up-regulation of NADPH oxidase 4 (Nox4) contributes to the activated phenotype of HGF expressing fibroblasts, via promoting oxidative stress. Scavenging ROS with an SOD mimetic, MnTE-2-PyP significantly suppressed the HGF-stimulated 3D growth and invasion of MDA-MB231 cells. In addition, targeting the ROS-generating Nox4 enzyme inhibited the ability of activated fibroblasts to contract collagen matrix. Our *in vivo* study further showed that scavenging ROS with EcSOD in MDA-MB231 significantly inhibited HGF-mediated tumor growth in mice. Overall, our study indicates a contributing role of an oxidative tumor microenvironment in promoting an activated-phenotype of fibroblasts in addition to supporting HGF/c-Met signaling during cancer-fibroblast interactions.

## RESULTS

### EcSOD inhibits c-Met phosphorylation

**Figure 1:**
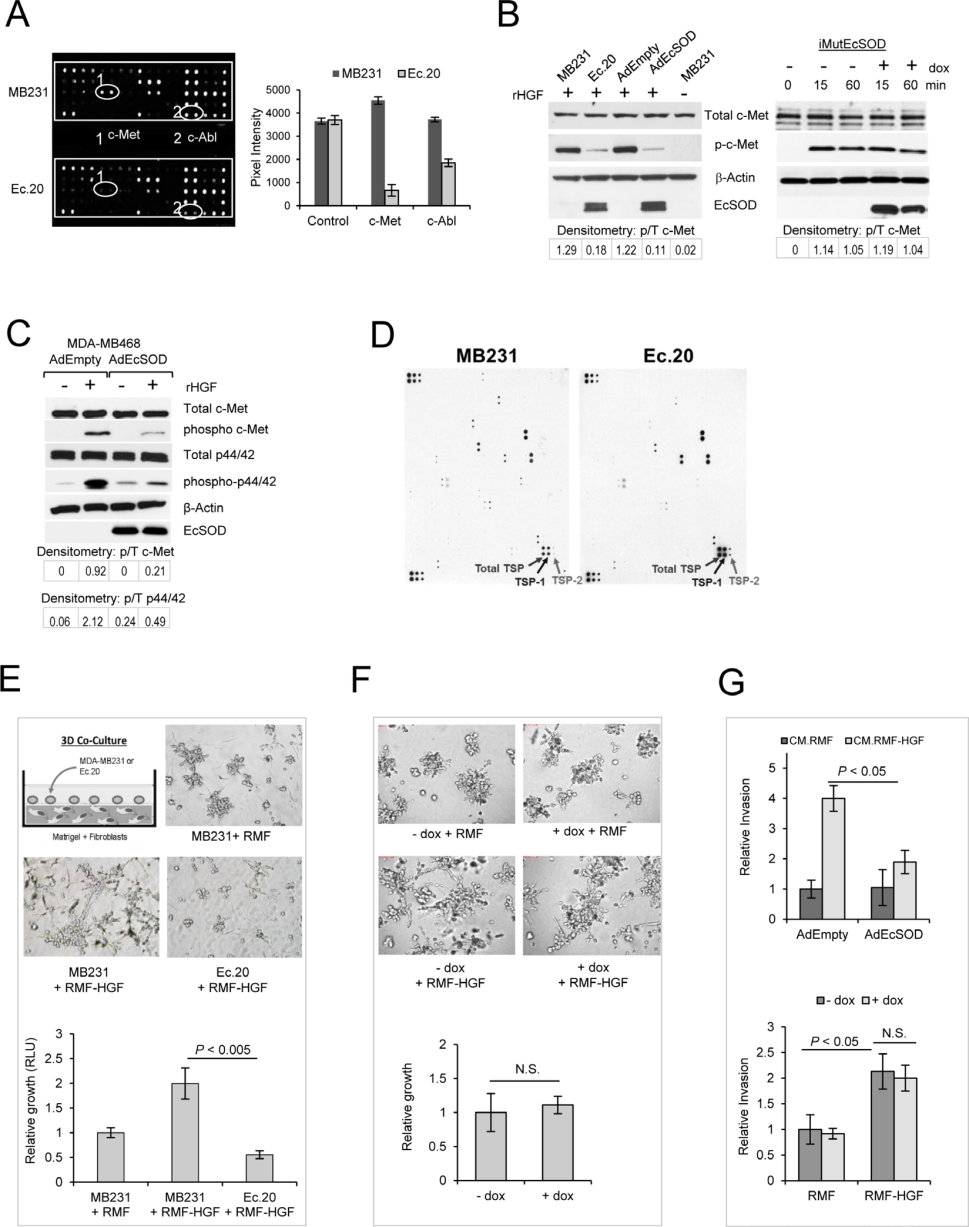
Overexpression of EcSOD inhibited c-Met phosphorylation and HGF-stimulated Matrigel growth of MDA-MB231 cells (**A**) Cell lysates of the parental MDA-MB231 or an EcSOD overexpressing stable cell line (Ec.20) were screened for activation of a panel of receptor tyrosine kinases using the PathScan^®^ RTK Signaling Antibody Array. A fluorescence image of the array chip revealing phosphorylation of c-Met and c-Abl in MDA-MB231 cells vs. EcSOD-overexpressing Ec.20 cells. Pixel intensities of the array signal is shown on the bar graph. (**B**) Left–western blot analysis confirming that either transient overexpression of EcSOD using adenovirus vectors (AdEcSOD) or stable overexpression in Ec.20 clones showed significant decreases in c-Met activation (phosphorylation at Y1234/1235). Cells were serum starved overnight followed by rHGF stimulation (50 ng/mL) for 15 min prior to cell lysate preparation. Right–Overexpression of inactive mutant EcSOD (N180A, R186A) did not affect c-Met activation in MDA-MB231 cells. Doxycycline (100 ng/mL) was used to induce the expression of the mutant EcSOD in iMutEcSOD cells. Cells were serum starved overnight followed by HGF stimulation (50 ng/mL) for 15 and 60 min prior to cell lysate preparation. Signal intensity of phosphorylated c-Met over total c-Met was analyzed by densitometry as shown. (**C**) Phosphorylation of c-Met (Y1234/1235) was also inhibited by EcSOD in MDA-MB468 cells. Cells were infected with AdEcSOD or AdEmpty for 24 hours prior to stimulation with 50 ng/mL of rHGF. Cell lysate was then harvested for western blot analysis after 15 min of stimulation. Representative blots from 3 independent experiments are shown. Signal intensity of phosphorylated c-Met over total c-Met and phosphorylated p44/42 over total p44/42 was analyzed by densitometry as shown. (**D**) Extracellular protein array analysis comparing the secreted factors in Ec.20 cultured media to that of the vector control MDA-MB231 cells. (**E**) EcSOD inhibited HGF-stimulated invasive morphology and growth in 3D co-culture. Top–Mammary fibroblasts (RMF or RMF-HGF) were embedded in Matrigel and breast cancer cells (MDA-MB231 or Ec.20) were seeded on top of the matrix as illustrated in the top panel. Bright field images showing the 3D growth of MDA-MB231 or Ec.20 cells when co-cultured with RMF-HGF for 4 days. Representative images of *N* = 3 separate experiments. Bottom–Cellular growth of breast cancer cells was quantified after 4 days of co-culture with a luciferase activity assay. RLU = Relative Light Units. Representative data from *N* = 3 separate experiments. Error bars = SD of 6 separate samples. (**F**) Catalytically inactive EcSOD did not affect the 3D morphology and growth of iMutEcSOD cells. Top–3D growth of iMutEcSOD in co-culture with RMF or RMF-HGF. 100 ng/mL of dox was used to induce the mutant EcSOD expression. Bottom–After 5 days of culture, cells were isolated from the matrix and counted for growth analysis. Error bars = SD of 4 separate samples. (**G**) Wild-type EcSOD but not the inactive mutant (N180A, R186A) inhibited breast cancer cells invasion, under the stimulation of RMF-HGF. Top–Representative Matrigel invasion of MDA-MB468 cells when EcSOD was overexpressed with AdEcSOD versus AdEmpty. Conditioned media (CM) harvested from fibroblasts (RMF and RMF-HGF) were used as chemoattractant. Invaded cells were analyzed after 20 hours of seeding. Data are mean ± SD of 3 separate samples. Bottom–Representative invasion of iMutEcSOD when the expression of an inactive mutant EcSOD was induced with 100 ng/mL of dox, in the presence of CM harvested from RMF and RMF-HGF. Data are mean ± SD of 3 separate samples.

To determine if scavenging cell surface-generated ROS with EcSOD will affect the phosphorylation status of RTKs, we utilized PathScan^®^ RTK Signaling Antibody Array Kit (Cell Signaling). As shown in Figure 1A, amongst the 28 RTKs screened, the most significant change is observed for c-Met phosphorylation (Pan-Tyr), where overexpression of EcSOD (Ec.20 cell line) drastically inhibited activation of this RTK relative to its vector control MDA-MB231 cell line. Quantification of the pixel intensities is shown on the right bar graph (A), revealing a more than 85% decrease in c-Met phosphorylation in Ec.20 cells versus MDA-MB231. The array shown in Figure 1A also shows a down-regulation of c-Abl phosphorylation (pan-Tyr), a non-receptor tyrosine kinase which has recently been shown to be activated downstream of c-Met [16–18]. To further confirm that overexpression of EcSOD affects c-Met activation in MDA-MB231, we performed western blot analysis. Figure 1B shows that when cells were stimulated with a recombinant human HGF (rHGF), c-Met phosphorylation (Y1234/1235) was significantly suppressed in Ec.20 cell line when compared to that of MDA-MB231 cells. Moreover, transient overexpression of this extracellular antioxidant using an adenovirus vector (AdEcSOD) at M.O.I. of 50 also resulted in an inhibition of c-Met activation relative to the control vector (AdEmpty) infected sample. To determine whether the superoxide scavenging activity of EcSOD is required for this inhibition, we overexpressed an inactive form of EcSOD where two critical residues were mutated as previously described [19]. The inactive EcSOD expression was generated to be expressed only under doxycycline induction and the cell line was named iMutEcSOD. When iMutEcSOD cells were treated with doxycycline, we observed no alterations in c-Met signaling as shown on the right panel in Figure 1B. These data suggest that HGF/c-Met signaling is sensitive to ROS modulation. To further show that the inhibitory effect of the wild type EcSOD on c-Met is not limited to MDA-MB231 cell line, we repeated the assay with another basal-like breast cancer cell line, MDA-MB468 and demonstrated similar down-regulation of this signaling pathway (Figure 1C).

### EcSOD up-regulates extracellular thrombospondin levels

To further determine whether EcSOD mediates suppression of c-Met signaling via an alteration of secreted factors, we performed an extracellular protein array analysis. As shown in Figure 1D, levels of thrombospondin-1 and 2 (TSP-1 and TSP-2) were significantly increased in conditioned media of Ec.20 compared to that of the vector control cells. Both TSP-1 and TSP-2 are anti-angiogenic and anti-metastatic factors that show inverse correlation with malignant progression in breast cancer [20, 21]. TSP-1 possesses a strong affinity for HGF [22] which mobilizes HGF away from the extracellular matrix and cell surface proteoglycans hence preventing its receptor binding [23]. Our results suggest that EcSOD inhibits c-Met activation partly through sequestering of its ligand via anti-angiogenic TSPs.

### EcSOD inhibits HGF-mediated breast cancer invasive morphology and growth in 3D culture

Since tumor stroma cells, such as activated fibroblasts are the predominant source of HGF, we determined if EcSOD will affect the cancer cell-fibroblast interactions in the context of HGF stimulation. Here, we co-cultured MDA-MB231 cells with mammary fibroblasts that were generated to overexpress HGF (RMF-HGF) [24]. The parental fibroblasts, RMF are non-malignant fibroblasts isolated from reduction mammoplasty as described [24]. The 3D co-culture system (Figure 1E) shows that RMF-HGF greatly stimulated the growth and stellate formation of MDA-MB231 relative to the cells co-cultured with RMF. This stimulatory effect of RMF-HGF was inhibited in the presence of EcSOD, where Ec.20 cells showed stunted 3D growth with minimal stellate formation when co-cultured with RMF-HGF. Growth of MDA-MB231 and Ec.20 cells under the RMF-HGF stimulation was quantified using a luciferase activity assay and presented on a bar graph in Figure 1E. The inactive mutant form of EcSOD on the other hand, did not affect the 3D stellate formation nor the growth of MDA-MB231, in the presence of HGF, as shown in Figure 1F. Moreover, overexpression of the wild type EcSOD with an adenovirus vector (AdEcSOD) suppressed the HGF-stimulated Matrigel invasion of MDA-MB468 cells (Figure 1G-top panel), whereas the mutant EcSOD has no influence on the invasiveness of breast cancer cells, when conditioned media harvested from RMF-HGF fibroblasts were used as a chemoattractant (Figure 1G-bottom panel).

### Prolonged co-culture with EcSOD overexpressing breast cancer cells attenuated pro-invasive phenotype of RMF-HGF

**Figure 2:**
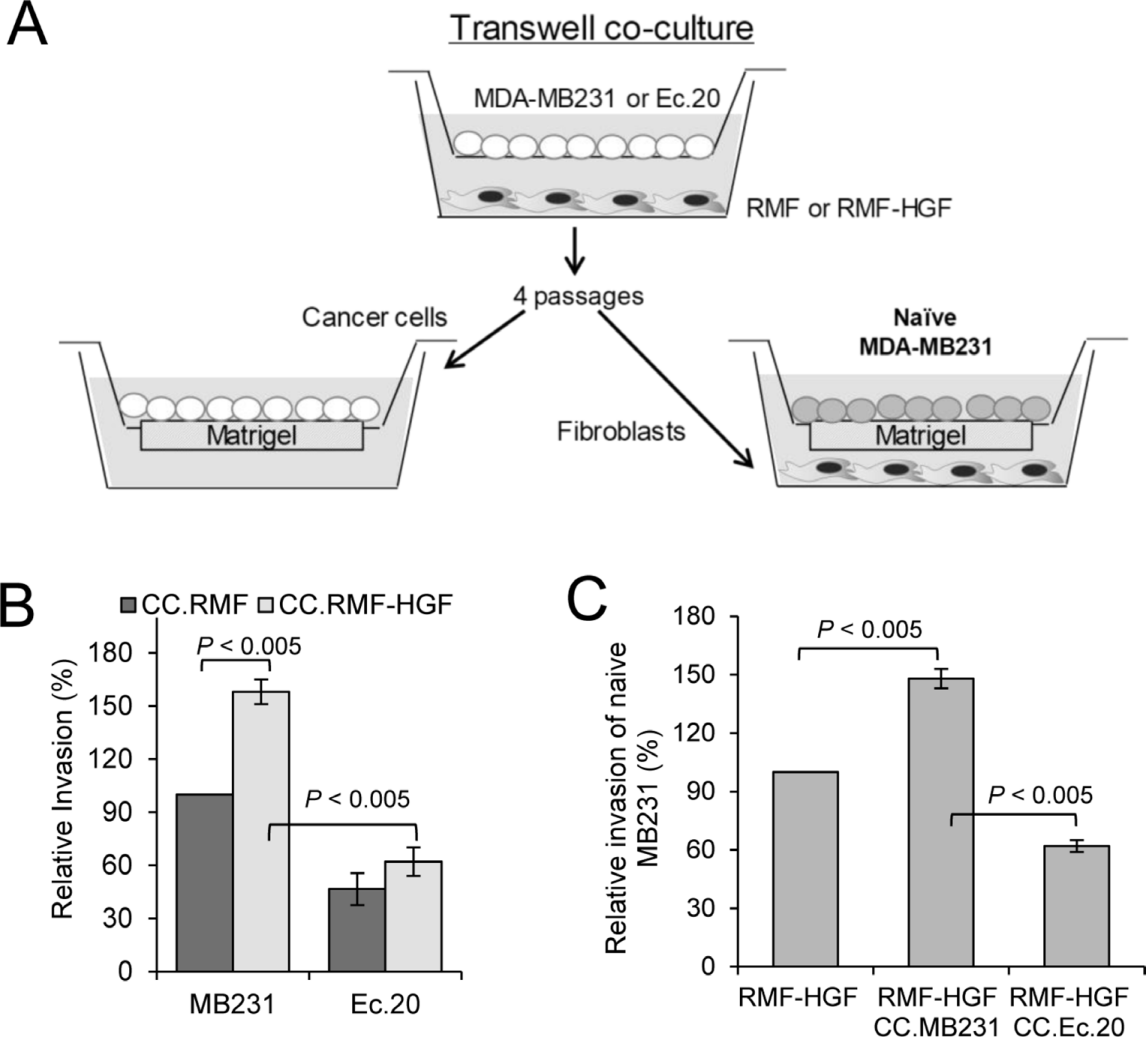
Prolonged co-culture with Ec.20 breast cancer cells inhibits pro-invasive phenotype of RMF-HGF (**A**) Breast cancer cells were in-directly co-cultured with RMF or RMF-HGF for 4 passages using transwell chambers. Breast cancer cells were subsequently isolated and evaluated for their invasiveness through Matrigel as shown in (**B**). Fibroblasts were also isolated and examined for their ability to promote invasion of naïve MDA-MB231 cells (**C**). Error bars = SD of 3 separate samples, representative data from *N* = 3 separate experiments.

To determine the effects of scavenging extracellular ROS with EcSOD on the reciprocal interactions between cancer cells and activated fibroblasts, we performed serial passages of MDA-MB231 cells with mammary fibroblasts using a transwell co-culture chamber as illustrated in Figure 2A. After four passages, fibroblast-co-cultured MDA-MB231 and Ec.20 cells were isolated and tested for their invasiveness through Matrigel. Figure 2B shows that as expected, MDA-MB231 cells are more invasive after prolonged co-cultivation with RMF-HGF (CC.RMF-HGF) when compared to co-cultivation with RMF (CC.RMF). In contrast, invasiveness of Ec.20 cells was not affected by the pro-longed interactions with RMF-HGF (CC.RMF-HGF), suggesting that EcSOD inhibits HGF-mediated invasion of cancer cells.

In parallel, RMF and RMF-HGF were also isolated from the co-cultures and tested for their ability to promote breast cancer cell invasion. As shown in Figure 2A, fibroblasts were seeded into the bottom wells of invasion chambers where naïve MDA-MB231 cells were seeded on the Matrigel layer on the top inserts. The bar graph in Figure 2C shows that RMF-HGF that were pre-educated by MDA-MB231 in this co-culture system (CC.MDA-MB231) became more aggressive in their ability to promote breast cancer cell invasion when compared to the fibroblasts that have not been co-cultured with the cancer cells. This shows a reciprocal pro-oncogenic stimulation between the fibroblasts and the breast cancer cells. However, RMF-HGF that were co-cultured with Ec.20 cells (CC.Ec.20) exhibited suppressed ability of this process. These data imply that ROS are involved in regulating the reciprocal cancer cell-fibroblast interactions in the context of HGF/c-Met activation.

### HGF expressing fibroblasts exhibit higher levels of ROS and an up-regulation of NADPH oxidase 4

**Figure 3:**
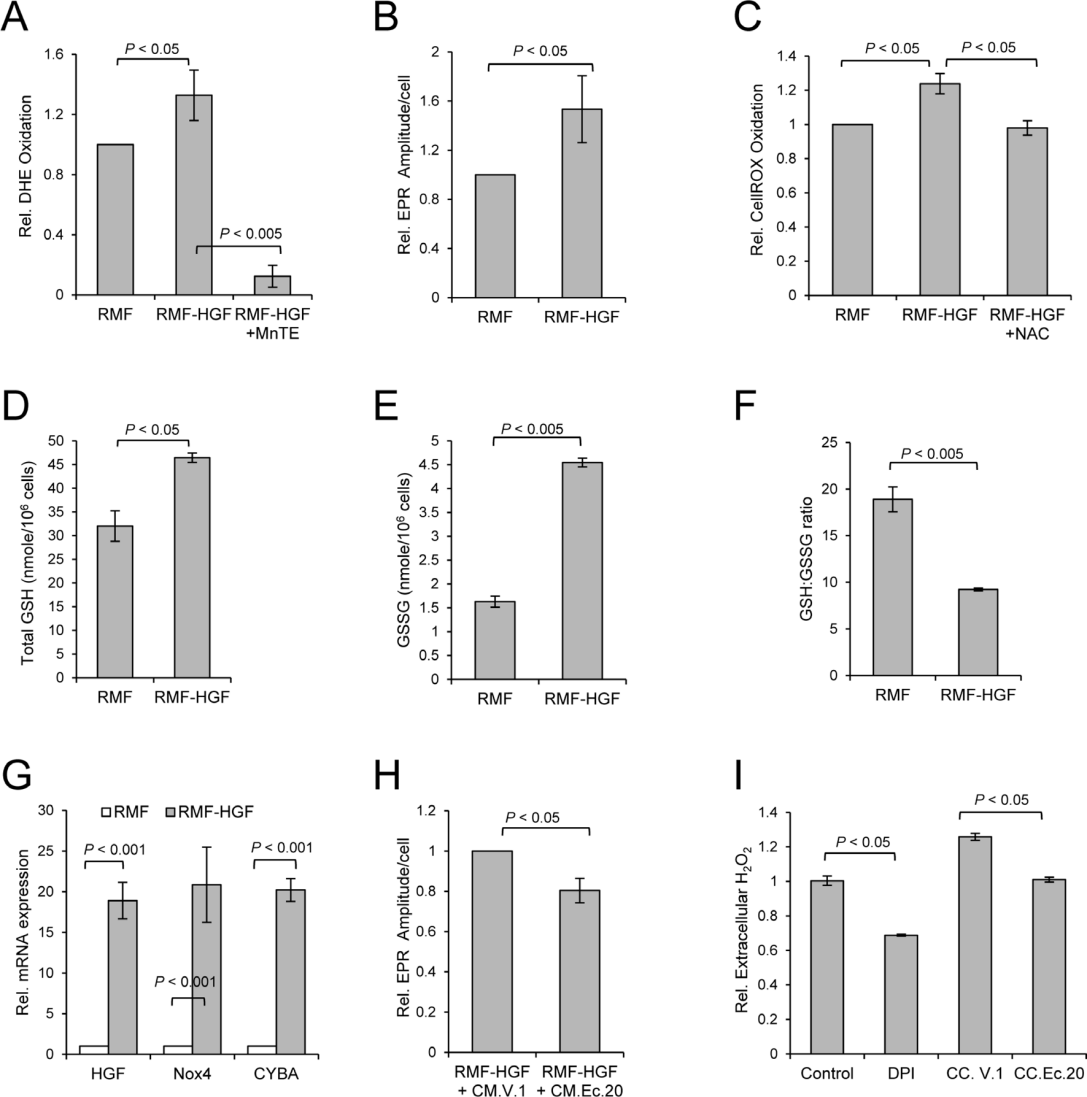
RMF-HGF fibroblasts generate higher levels of ROS in comparison to RMF (**A**) Intracellular O_2_^•−^ level was quantified with DHE by FACS analysis in fibroblasts. Cells were treated with 15 uM of MnTE for 16 h prior to DHE labeling. Data are mean ± SD of 3 separate samples, representative data from *N* = 3 independent studies. (**B**) Representative electron paramagnetic resonance (EPR) spectroscopy analysis of O_2_^•−^ using a superoxide-specific probe, 1-hydroxy-3-methoxycarbonyl-2,2,5,5-tetramethylpyrrolidine (CMH). Data are mean ± SD of 3 separate samples. (**C**) ROS level was determined using CellRox reagent. Fibroblasts were treated with NAC (5 mM) for 16 h prior to labeling cells with CellRox reagent. Representative data from *N* = 3 independent studies. Data are mean ± SD of 3 separate samples. (**D**–**F**) RMF-HGF show significantly increased levels of both the total GSH and the oxidized form, GSSG, when compared to RMF, resulting in a reduction in the GSH:GSSG ratio. Data are mean ± SD of 3 independent experiments. (**G**) Real time-PCR analysis shows an up-regulation of Nox4 and CYBA in RMF-HGF vs. RMF fibroblasts. Data are mean ± SD of 3 independent experiments. (**H**) Representative superoxide-specific EPR analysis of RMF-HGF. Fibroblasts were exposed to CM harvested from Ec.20 versus vector control MDA-MB231 cells (V.1) for 48 hours. Data are mean ± SD of 4 separate samples. (**I**) Extracellular H_2_O_2_ levels as determined by Amplex Red assay. RMF and RMF-HGF were co-cultured (CC) with V.1 or Ec.20 cells for 48 prior. RMF-HGF were also treated with 10 μM of diphenyleneiodonium (DPI), a pan Nox and flavoprotein inhibitor, as a control. Data are mean ± SD of 3 separate samples.

Since RMF-HGF shows an attenuated phenotype after the prolonged co-culture with Ec.20 cells (Figure 2C), we next determined if this oncogenic cytokine promotes oxidative stress in the activated fibroblasts. Figure 3A shows an increase in DHE oxidation in RMF-HGF when compared to RMF, indicating a higher level of O_2_^•−^ generation in the HGF expressing fibroblasts. As expected, DHE oxidation decreased when RMF-HGF were treated with an SOD mimetic, MnTE. In addition to performing the DHE oxidation assay using a more superoxide-specific setting at 405 nm excitation (instead of the non-specific 488 nm excitation) and 570 nm emission, as described [25], we have further determined the O_2_^•−^ levels in the fibroblasts with electron paramagnetic resonance (EPR) spectroscopy. As shown in Figure 3B, RMF-HGF indeed possess ~53% increase in superoxide-specific EPR amplitude per cell, as compared to RMF. We also utilized another oxidative stress indicator, CellROX® reagent, which is sensitive to ROS modifications. An increase in CellROX oxidation further shows that HGF overexpression promoted generation of ROS in mammary fibroblasts and this increase in ROS was reduced when cells were treated with an H_2_O_2_ scavenger, NAC (Figure 3C). In addition, cellular glutathione (GSH) levels were also analyzed as an overall indicator of cellular redox status. GSH is the most abundant antioxidant in aerobic cells and a decrease in the ratio of reduced to oxidized form (GSH:GSSG) indicates an increase in cellular oxidative stress, as often detected in cancer cells. We found that not only is GSH generation increased in RMF-HGF (Figure 3D), these fibroblasts also possess a close to 3-fold higher level of oxidized GSH, GSSG (Figure 3E), resulting in a significantly reduced GSH:GSSG ratio (Figure 3F), when compared to that of RMF. Taken together, these results indicate an increased oxidative stress in RMF-HGF versus RMF, due in parts to accumulation of O_2_^•−^ and other ROS.

To gain insight into the mechanisms that promoted oxidative stress in RMF-HGF versus its parental fibroblast strain, RMF we profiled mRNA expression of a small panel of antioxidant related genes in these fibroblasts using TaqMan^®^ Array Human Antioxidant Mechanisms kit. We found that CYBA (also known as p22phox) is highly up-regulated in RMF-HGF when compared to the RMF. CYBA is an essential component of the membrane-associated enzyme NADPH-oxidases (Noxs), more specifically Nox1-4. An increase in expression of CYBA suggests that activation of Noxs is one of the contributing factors in promoting oxidative stress in RMF-HGF. We have indeed confirmed that there is a 20-fold increase in Nox4 mRNA expression in RMF-HGF versus RMF (Figure 3G), while no significant difference was observed in the mRNA expression levels of Nox1-3 (data not shown). This suggests that Nox4 is the predominant source of ROS in HGF-overexpressing fibroblasts.

To show the effect of cancer-fibroblast co-culture on cellular redox status of the fibroblasts, we determined the O_2_^•−^ levels in RMF-HGF when cultured in the presence of conditioned media harvested from Ec.20 breast cancer cells. Figure 3H shows that there was a significant reduction in superoxide-specific EPR amplitude in these fibroblasts in the presence of EcSOD-containing media (CM.Ec.20) versus vector control media (CM.V.1). Since H_2_O_2_ can cross membrane, we also measured the extracellular levels of this peroxide as an indirect indicator of the cellular H_2_O_2_ levels. Figure 3I shows that the extracellular H_2_O_2_ levels of RMF-HGF were also significantly reduced, when co-cultured with Ec.20 breast cancer cells. These results suggest that exogeneous sources of EcSOD can regulate cellular redox status of RMF-HGF.

### Nox4 promotes collagen contraction activity of fibroblasts

**Figure 4:**
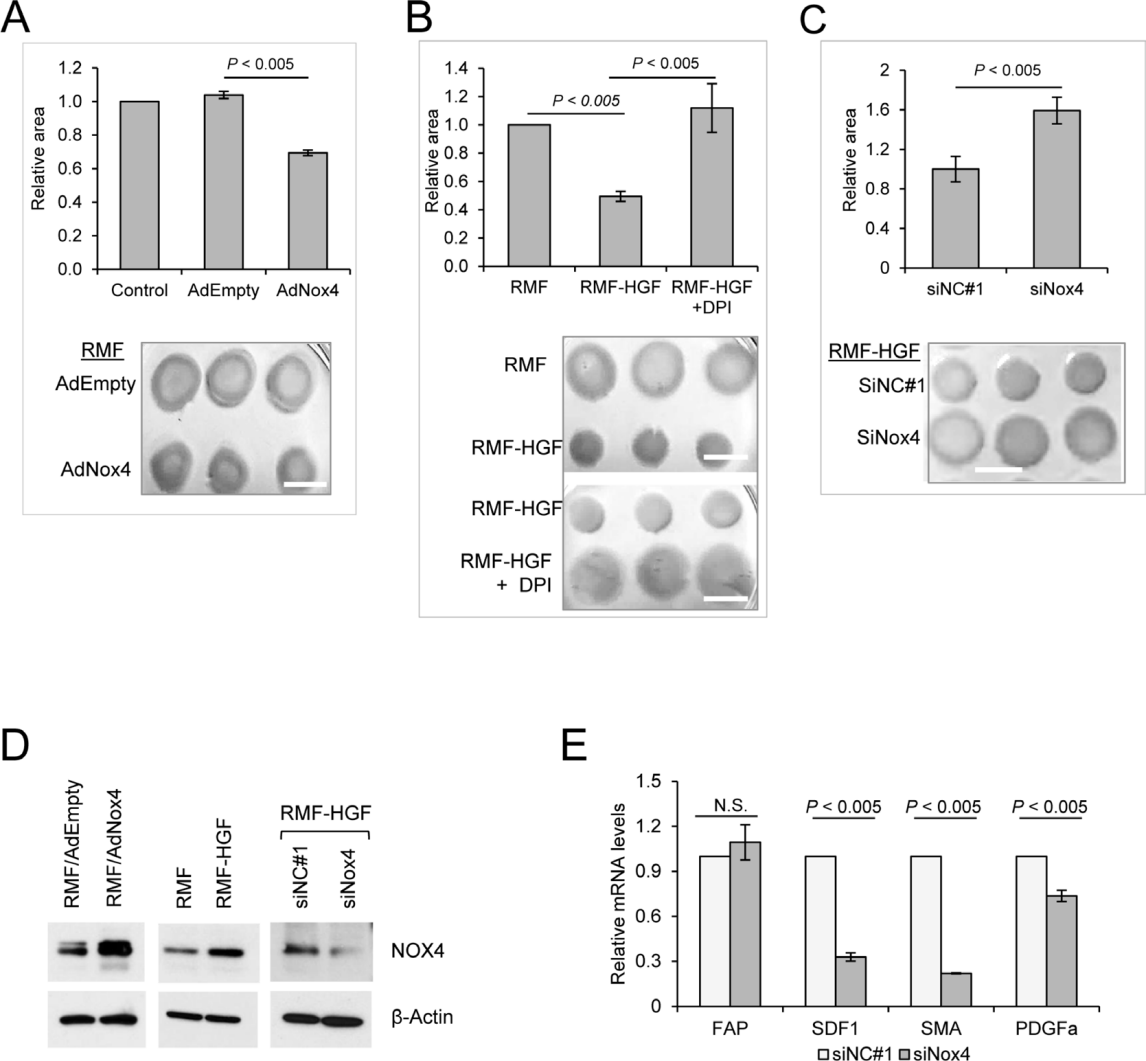
Nox4 promotes collagen gel contraction ability of fibroblasts (**A**) RMF was induced to overexpress Nox4 with adenovirus vector (AdNox4 vs AdEmpty as a control). Collagen contraction activity of fibroblasts was then analyzed after 16 h of seeding the cells, where area of contracted collagen discs were quantified by Image J. The effect of Nox4 on contraction activity of RMF-HGF was determined by (**B**) inhibiting the ROS-generating activity of Nox4 with DPI and by (**C**) suppressing expression of Nox4 using an siRNA (siNox4 vs siNC#1 as a control). White bar marks 1cm in length. Representative images from *N* = 3 independent collagen contraction experiments. Error bar = SD of 3 separate samples. (**D**) Western blot analysis showing the expression levels of Nox4 in fibroblasts. Representative images from 3 independent experiments. (**E**) Nox4 knockdown in RMF-HGF down-regulates mRNA expression of myofibroblast markers. Real time PCR analysis of siRNA transfected RMF-HGF. Error bar = SD of 3 separate experiments.

To determine the effects of Nox4-generated ROS on the activity of fibroblasts, we performed collagen contraction assays where Nox4 was overexpressed in RMF using an adenovirus vector (AdNox4). This 3D collagen gel contraction is a standard assay utilized for functionally quantifying a contractile phenotype of activated fibroblasts. Figure 4A shows that the control adenovirus vector (AdEmpty) infected RMF showed similar contraction rate compared to the mock infected control sample. However, in the presence of Nox4, a reduction in the collagen disc area was observed, indicating an increase in collagen contraction activity of AdNox4 infected RMF.

Figure 4B shows that when compared to RMF, HGF-overexpressing fibroblasts showed a significant increase in collagen contraction, indicating a more activated and aggressive phenotype of the RMF-HGF. In the presence of a Nox inhibitor DPI, RMF-HGF showed an attenuated ability to contract collagen. This suggests that ROS-generating Nox enzymes contribute to fibroblast activation. Since DPI targets the activity of all Noxs, we specifically inhibited Nox4 expression in RMF-HGF using an siRNA and observed that down-regulation of Nox4 also significantly impaired the ability of RMF-HGF to contract collagen (Figure 4C). Protein expression levels of Nox4 in these fibroblasts were verified using western blot analysis as shown in Figure 4D. In addition to collagen gel contraction, we also evaluated the mRNA expression levels of some myofibroblast markers in Nox4-knocked down fibroblasts. Figure 4E shows that transfection with siNox4 down-regulated expression of stroma derived factor 1 (SDF1), smooth muscle actin (SMA), and platelet derived growth factor receptor alpha (PDGFRa) in RMF-HGF when compared to the control siNC#1 transfected cells. Together, this study indicates that Nox4 up-regulation in RMF-HGF promotes an activated phenotype of the fibroblasts.

### Scavenging ROS with an SOD mimetic inhibited HGF-mediated growth and invasion of breast cancer cells

**Figure 5:**
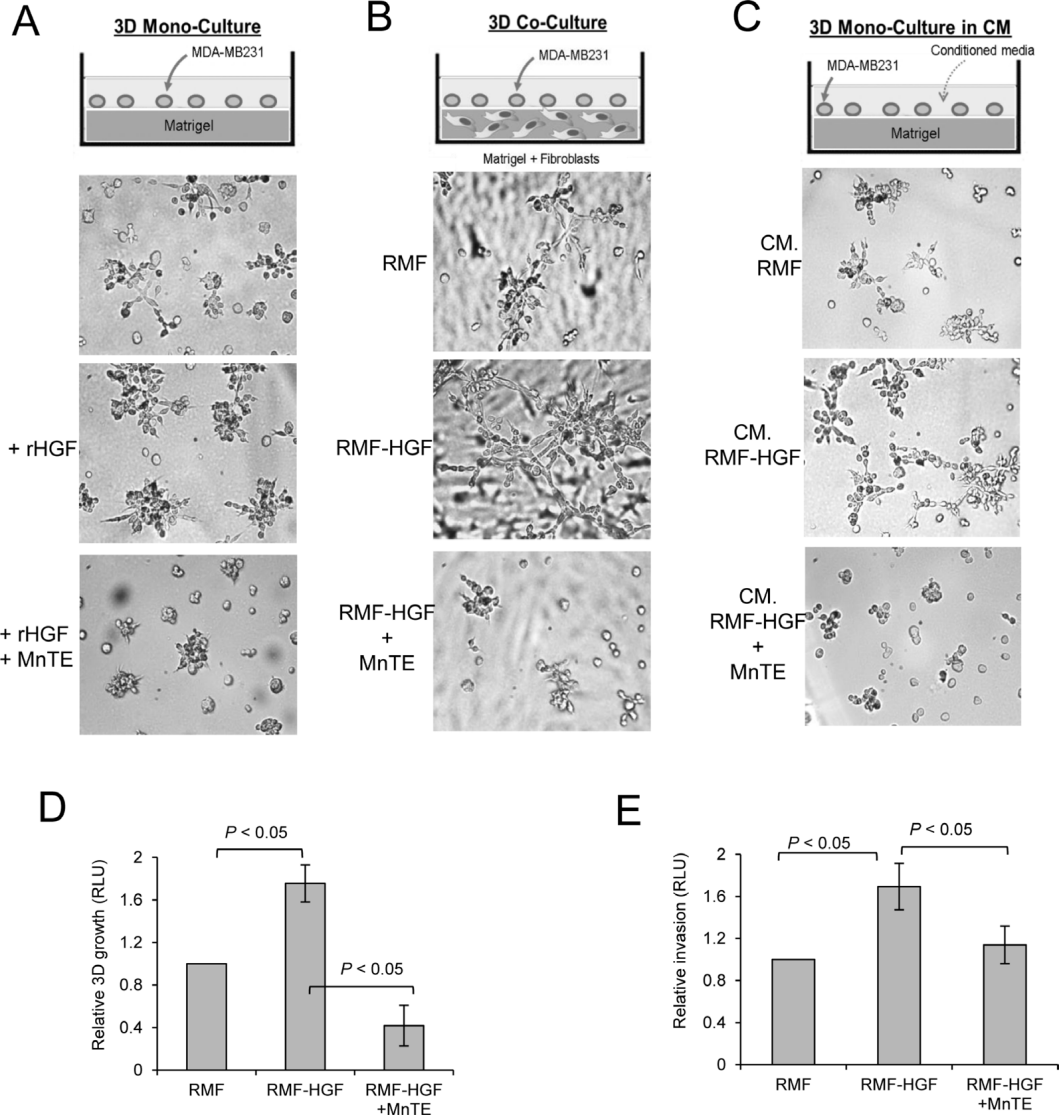
Scavenging ROS with an SOD mimetic inhibited HGF-mediated 3D growth and Matrigel invasion of MDA-MB231 Growth of MDA-MB231 cells was stimulated with either rHGF (50 ng/mL, **A**) or in co-culture with RMF-HGF (**B**), or with conditioned media (CM) harvested from fibroblasts (C). Cells were treated with an ROS scavenger, MnTE-2-PyP (MnTE, 15 uM) for 4 days. Representative bright field images of *N* = 3. Quantitation of the proliferation of MDA-MB231 in (**C**) is shown in (**D**). After 4 days of culture with conditioned media from RMF or RMF-HGF, MDA-MB231 cells were lysed and luciferase activity was measured. (**E**) MDA-MB231 cells were seeded into Matrigel^®^ invasion chamber and their ability to invade into conditioned media harvested from RMF or RMF-HGF in the present and absence of MnTE (15 uM) was analyzed. *N* = 3 independent studies. Data are mean ± SD of 3 separate samples. RLU = Relative light units.

To further provide evidence that ROS contributes to HGF-mediated oncogenic stimulation, we treated breast cancer cells with an SOD mimetic, MnTE-2-PyP (MnTE). This compound has been demonstrated to effectively scavenge ROS [26]. We first stimulated 3D growth of MDA-MB231 with rHGF and observed that MnTE treatment inhibited the induction of breast cancer cell growth and stellate formation by HGF (Figure 5A). Next, we co-cultured breast cancer cells with RMF-HGF. In this co-culture, MnTE also greatly reduced the 3D colony sizes and invasive stellate formation of MDA-MB231 as seen in Figure 5B. Moreover, this inhibitory effect of MnTE is observed when cancer cells were stimulated with conditioned media harvested from RMF-HGF (Figure 5C). Cellular growth in conditioned media (shown in Figure 5C) was quantified using a luciferase activity assay and presented in Figure 5D. The antioxidant, MnTE inhibited HGF-mediated growth of breast cancer cells by 76% (*P* = 0.017). In addition to the 3D growth behavior, we also determined the effects of scavenging ROS with MnTE on HGF-mediated invasion of breast cancer cells. Figure 5E shows that while conditioned media from RMF-HGF promoted invasion of MDA-MB231, this stimulation was significantly reduced in the presence of MnTE. These studies reveal that HGF-mediated cancer cell-fibroblast interactions involve ROS and that administration of antioxidants can suppress the oncogenic HGF/c-Met signaling.

### EcSOD inhibited HGF-mediated tumor growth

**Figure 6:**
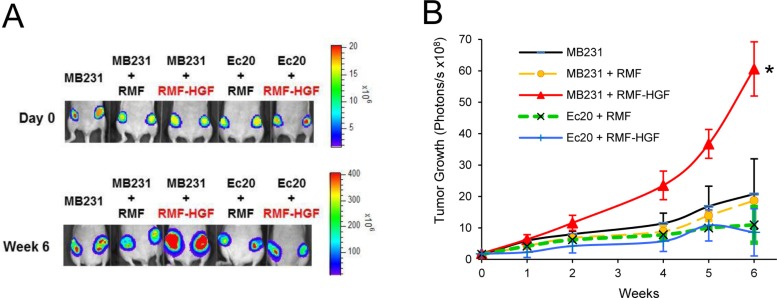
EcSOD suppresses HGF-stimulated tumor growth (**A**) Bioluminescence imaging showing tumor growth of luciferase expressing breast cancer cell lines in athymic nude mice. At Day 0, 10^6^ MDA-MB231 or Ec.20 cells were co-injected with 3 × 10^5^ fibroblasts (RMF or RMF-HGF) into both sides of the 4^th^ mammary fad pads. Mice were imaged over time to monitor tumor growth as shown in representative images in (A) and the quantitation of the primary tumor growth is shown in (**B**). *N* = 6 per group. ^*^P < 0.01 Ec.20 + RMF-HGF vs. MB231 + RMF-HGF.

To determine the regulation of HGF-stimulated tumorigenesis by ROS, we utilized an orthotopic mammary tumor model. RMF-HGF was co-injected with the cancer cells as the source of c-Met ligand as well as in providing a model for studying the tumor-fibroblast interactions. The top panel of Figure 6A shows the presence of the breast cancer cells following the mammary fat pad injection. After 6 weeks post injection, RMF-HGF greatly stimulated the primary tumor growth of MDA-MB231, as expected (bottom panel). In contrast, EcSOD overexpressing cells (Ec.20) were irresponsive to the growth stimulation by RMF-HGF. Quantitation of the primary tumor growth is presented in Figure 6B as determined by photon flux over time.

### EcSOD is significantly under-expressed in breast carcinomas

**Figure 7:**
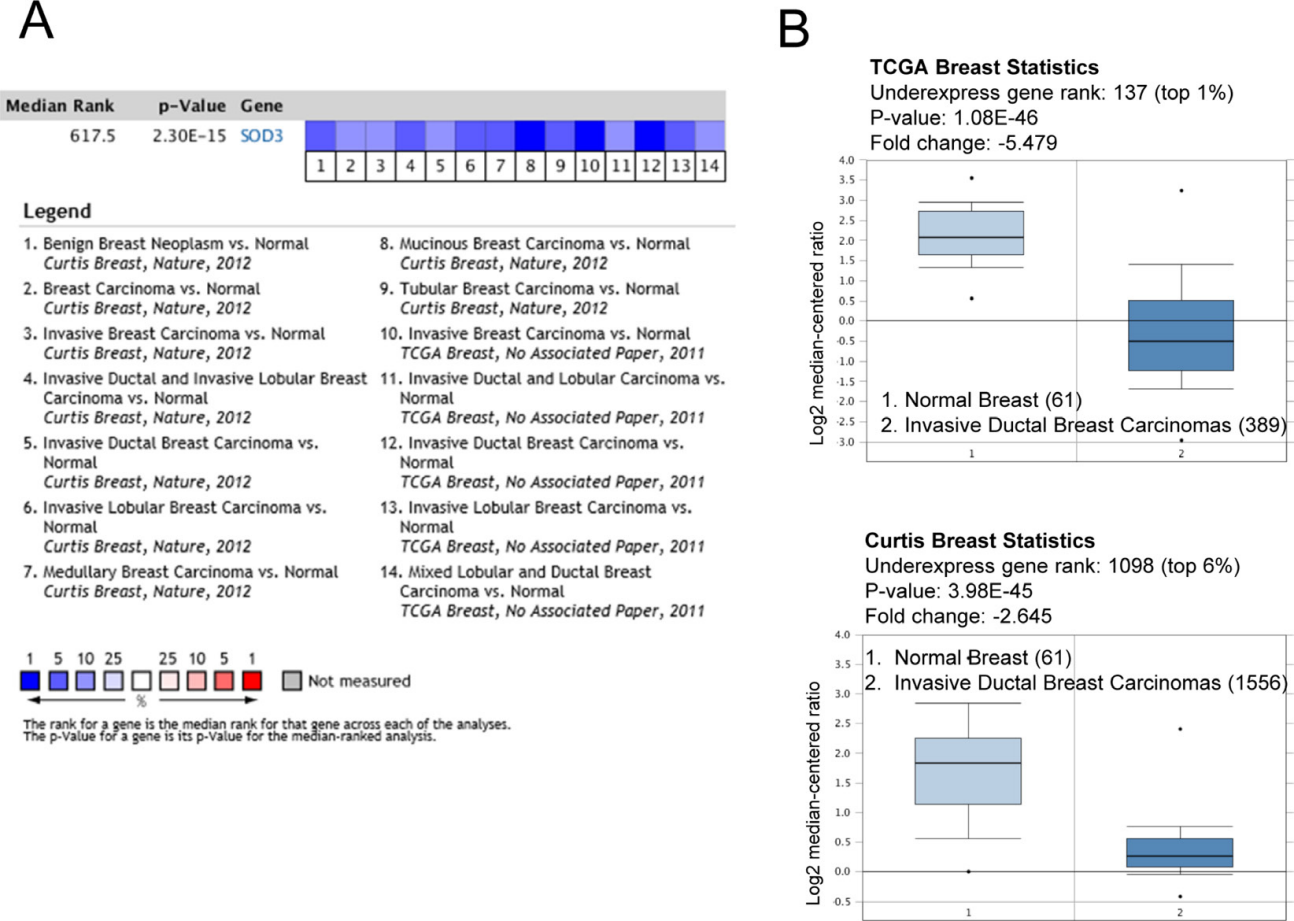
Oncomine SOD3 (*EcSOD*) gene analysis in breast cancer (**A**) Comparison of SOD3 expression across 14 breast cancer analyses. The heatmap represent the relative expression in patients with the indicated breast carcinomas compared with normal tissue. Blue indicates underexpression. The reported median rank and *P* value consider all indicated studies simultaneously. (**B**) Box plots derived from gene expression data comparing invasive ductal carcinomas to normal tissues from TCGA and Curtis studies. The number of samples in each group is indicated in brackets.

To further evaluate the expression levels of EcSOD in breast carcinomas, we analyzed deposited Oncomine datasets. Differential expression analysis of breast carcinomas versus normal tissues showed that EcSOD (or SOD3) is significantly down-regulated in different types of breast carcinomas in two main datasets, TCGA and Curtis Breast (Figure 7A). Specifically, in invasive breast carcinomas, SOD3 is ranked in the top 1% and 6% most significantly under-expressed genes as shown in the box plots in Figure 7B. The fold change of this gene is −5.475 in the TCGA and −2.654 in the Curtis datasets.

### Low EcSOD expression confers poor survival in breast cancer patients

An integrative microarray data analysis using the Kaplan Meier Plotter [27] shows that low EcSOD expression is associated with significantly reduced relapse free survival in all breast cancer subtypes, including the basal-like (ER−, PR−, Her2−, CK 5/6+, and/or EGFR+) breast cancers (Table 1). Survival analysis was performed where restriction was set to exclude systemically untreated patients. The numbers of samples in each group are indicated in parentheses, and the hazard ratios (HR) and log rank *p* values are shown.

**Table 1:** Relapse free survival rate of breast cancer patients based on low versus high expression levels of EcSOD analyzed by Kaplan-Meier Plotter

	Low expression cohort (No. of patients)	High expression cohort (No. of patients)	Hazard Ratios (HR)	*P* values
All subtypes	61^*^ months (469)	173.2^*^ months (1412)	0.46	1.0E-16
Luminal A	42 months (223)	97 months (638)	0.48	2.9E-08
Luminal B	43.9 months (149)	171.4 months (447)	0.42	4.0E-11
Her2+	16 months (33)	22.6 months (92)	0.57	5.3E-02
Basal-like	11.31 months (105)	30.42 months (194)	0.46	1.7E-05

Gene expression data and survival information are downloaded from GEO (Affymetrix HGU133A and HGU133+2 microarrays), EGA and TCGA (http://kmplot.com).

Months shown are upper quartile RFS rate except for ^*^ which indicates median survival.

## DISCUSSION

A breast cancer genome-wide association study (GWAS) of the NCI Cancer Genetic Markers of Susceptibility project identified c-Met signaling as the second highest ranked pathway that may contribute to breast cancer susceptibility [28]. Since breast cancer cells primarily rely on their stroma fibroblasts for the c-Met ligand, understanding the contributing factors that fuel this reciprocal communication between cancer cells and their stroma partners will therefore, help in identifying novel targets for c-Met driven cancer. In this study, we identified c-Met as one of the RTKs regulated by an extracellular antioxidant enzyme, EcSOD in basal-like breast cancer cells via modulating the cellular redox status. Expression of this enzyme has been shown to be suppressed in a variety of cancers, including breast cancer [3, 29, 30]. Conversely, up-regulation of EcSOD has been reported to inhibit both *in vitro* and *in vivo* growth as well as the oncogenic phenotype of breast cancer, prostate cancer, pancreas cancer, melanomas, and lung cancer [4, 29, 31–35], suggesting tumor suppressive effects of this antioxidant.

The oncogenic RTK, c-Met is one of the cell surface receptors known to be activated by ROS. Stimulation of lung cancer cells with exogenous ROS (i.e. H_2_O_2_) enhanced tyrosine phosphorylation of c-Met and activated its downstream signaling cascades in lung cancer cells [6]. In these same cells, an increase in ROS production was also observed when c-Met was activated by HGF treatment [6]. In addition, ROS-induced invasive activity of hepatoma cells has been demonstrated to be mediated through an autocrine/paracrine loop of HGF, where ROS directly augment mRNA expression of HGF [36]. Our study provides further evidence that a pro-oxidative environment is involved in activating and sustaining the oncogenic HGF/c-Met signaling and that re-expressing EcSOD in breast cancer cells inhibits HGF-stimulated oncogenesis. Further supporting an association of c-Met signaling with loss of EcSOD expression, a gene expression profiling study identified EcSOD as one of the genes that was down-regulated in both c-Met driven mouse liver tumors and human hepatocyte carcinomas, and that this change has a significant predictive power on overall and disease-free survival [37].

Although the second messenger role of ROS in cellular signaling is well recognized, it is not clear how EcSOD specifically regulates c-Met signaling amongst the other kinases screened in this study. Interestingly, phosphorylation of other RTKs such as EGFR, ERBB2, and FLT-3 have recently been shown to be promoted when EcSOD is overexpressed in thyroid cancer cells [38]. Despite increased phosphorylation of these RTKs, EcSOD overexpressing cells showed a reduced level of growth and migration signal transductions, through down-regulation of small GTPase regulatory genes [38]. The same study also showed a reduction of HGFR (or c-Met) phosphorylation in EcSOD enriched cells. The discrepancies between our RTK findings with the exception of c-Met phosphorylation could be due to specificity issues related to the cell type examined and possibly also influenced by the dose-dependent effect of this ROS scavenger, as suggested by the authors.

One mechanism by which EcSOD could inhibit c-Met signaling is by reducing bioavailability of its ligand HGF, via TSPs, as shown by our extracellular protein array analysis. Thrombospondin 1,TSP-1 is a well known anti-angiogenic protein that elicits a variety of cellular processes, including a recently discovered role in maintaining dormancy of disseminated tumor cells [39]. The TSP-1 interactome is still not fully defined but HGF has been identified to be one of its ligands [40]. We have shown here for the first time that EcSOD expressing cells secrete higher levels of TSP-1 and TSP-2 (Figure 1D). Not only is the function of HGF affected by TSP-1, the expression of this anti-angiogenic factor has also been shown to be down-regulated by HGF-c-Met interactions in breast cancer cells [41], indicating a negative regulatory circuit of TSP1 and HGF. Further supporting a regulatory effect of EcSOD on TSP-1, a microarray expression analysis has identified TSP-1 as one of the growth suppressors upregulated by this antioxidant in thyroid cancer cells [38].

It is now well recognized that the progression from normal to benign, malignant, and metastatic, as well as therapeutic resistance is driven not just by what is happening inside the cancer cell but by reciprocal communication between the cancer cells and their stroma microenvironment. Our heterotypic co-culture studies indicate an ROS-mediated reciprocal interaction between cancer cells and fibroblasts (Figure 2). After a prolonged co-cultivation with CAF-like RMF-HGF, breast cancer cells showed a heightened aggressiveness in invasive properties and they also pre-educated RMF-HGF to increase their ability to promote breast cancer invasion. This symbiotic relationship is compromised when breast cancer cell overexpress EcSOD. The presence of EcSOD in breast cancer cells not only inhibited this RMF-HGF-stimulated invasion, but on top of that prevented the reprograming of RMF-HGF in their tumor-promoting phenotype. This suggests that an oxidative microenvironment is a contributing factor in regulating HGF-c-Met-mediated tumor stroma co-evolution. This is particularly important considering the fact that 86% of the aggressive basal-like tumors are positively correlated with the HGF signature [42]. Furthermore, among basal-like patients that are positive for the HGF signature, patients had worse overall survival [42]. While some c-Met signaling can arise from an autocrine manner, the predominant activation mode of c-Met in breast cancer is through a paracrine tumor-stroma interactions. Immunohistochemistry analysis shows that the HGF/c-Met paracrine pattern is seen in 59.1% of tumors; and that this paracrine signaling is associated with a worse outcome when c-Met staining is more intense at the tumor front [43].

Cancer associated fibroblasts (CAFs) actively participate in the outcome of breast cancer by providing extracellular matrix components and secreting signaling factors that enhance cancer cell growth, survival, oncogenic progression, and metastasis. However, the intrinsic factor(s) contributing to the “activated” fibroblast phenotype of CAFs remains to be elucidated. A recent study showed that oxidative stress is necessary for triggering fibroblast activation in the pathological condition of fibrosis. NADPH oxidases (Noxs) are the predominant enzymes that produce O_2_^•−^ on the plasma (and organelle) membrane and their activation has been linked to the etiology of cancer. Importantly, Nox4 is the only Nox that is expressed as an active enzyme. Thus, elevated Nox4 will lead to an increase in ROS generation. Nox4 has been suggested to play an important role in inducing fibroblast activation during fibrosis [44] in cardiac and pulmonary fibroblasts and in TGFβ-induced myofibroblast activation [45], but the role of Nox4 in CAFs is not known. Here we have shown that the HGF-induced activated phenotype of mammary fibroblasts involves Nox4-generated ROS (Figures 3 and 4). This explains the suppressive effects of EcSOD during co-cultivation of breast cancer cells with RMF-HGF as well as in our *in vivo* tumor model. Modulating the redox tumor microenvironment will therefore, not only inhibit the oncogenic c-Met pathway in cancer cells, but also target the activated phenotype of CAFs, thus suppressing their tumor-stroma interactions.

Despite the name implying that EcSOD is an extracellular antioxidant, the O_2_^•-^ scavenging effect of this enzyme is not limited to the cell surface and the secreted compartment but also more widely distributed to endocytic vehicles and nucleus. By harboring a heparin binding domain at its c-terminus, EcSOD interacts with cell surface heparin sulfate proteoglycans [46] once it is secreted. This interaction has been shown to be critical for the re-entry of this enzyme through a clathrine-mediated endocytosis [47] and its nuclear translocation [48]. This feature renders EcSOD more readily available to cells or tissues that do not express this enzyme and internalization of exogenous EcSOD has been reported in mouse preadipocytes and endothelial cells [47, 48]. Here, the secreted EcSOD from Ec.20 cancer cells is also likely to be taken up by the co-cultured RMF-HGF, resulting in a decrease in O_2_^•−^ levels of the fibroblasts (Figure 3H), thereby attenuating the tumor-promoting effects of RMF-HGF, as seen in Figure 2.

Although Nox4 has been historically considered as a O_2_^•−^ generating enzyme, an increasing number of studies have reported that the major product of Nox4 is H_2_O_2_, although other studies have detected O_2_^•−^ generation [49–51]. It is plausible that some of the discrepancies may have resulted from non-specificity issues of the ROS detection reagents or that the O_2_^•−^ produced (in membrane compartments) remains cryptic and inaccessible to assay reagents prior to its dismutation to form H_2_O_2_. Since the oxygen-reducing heme group at the catalytic site of Nox4 is an obligate one-electron donor, a direct formation of H_2_O_2_ without a O_2_^•−^ intermediate is mechanistically implausible, implying that Nox4 is both a O_2_^•−^ and H_2_O_2_ producer. This is supported by a recent study using cell-free isolated Nox4, where the authors found that approximately 80% of the product from the isolated Nox4 was detected as H_2_O_2_, while ~20% was detected as O_2_^•−^ [49].

Since EcSOD catalyzes the dismutation of O_2_^•−^ to H_2_O_2_, some may argue that EcSOD would exacerbate the H_2_O_2_ levels in Nox4-overexpressing RMF-HGF, and if so overexpression of EcSOD would not have inhibited but promoted c-Met signaling, since this RTK is known to be activated by H_2_O_2_ [6]. Whether overexpression of SOD can increase the production of H_2_O_2_ has been a hotly debated issue. Although some studies have attributed the consequences of SOD overexpression to this counter intuitive effect in increasing H_2_O_2_ levels, the view that SOD should elevate H_2_O_2_ formation merely because it catalyzes an H_2_O_2_-producing reaction has been disputed. Liochev and Fridovich [52, 53] reasoned that an excess of SOD should decrease the steady-state levels of O_2_^•−^ without increasing the endogenous formation of H_2_O_2_, particularly *in vivo*. Therefore, it is doubtful that the inhibitory tumor-stroma effects seen with EcSOD overexpression in our current study, is due to an overproduction of H_2_O_2_. Moreover, the copper ion located in the catalytic domain of EcSOD are sensitive to H_2_O_2_ attack and by acting in a “suicidal mode”, EcSOD can have peroxidase-like properties [54]. Our data showing a decrease in both cellular O_2_^•−^ levels and the extracellular H_2_O_2_ levels in the co-cultures (Figure 3H and 3I) support the view that overexpression of this extracellular O_2_^•−^ scavenger does not contribute to H_2_O_2_ accumulation, but reduces cellular oxidative stress.

Although H_2_O_2_ has long been the main focus as the ROS-mediated signaling molecule, partly due to it being a more stable and longer lived species than O_2_^•−^ , O_2_^•−^ has recently been viewed as an important mediator of cellular effects. Superoxide affects both serine/threonine protein phosphatases (PPs) and protein tyrosine phosphatases (PTPs), by oxidizing the metal ion center of the former class of phosphatases and via nucleophilic attack of the cysteine residue in the later class [55, 56]. The rate of O_2_^•−^ signaling has been estimated to be about 10–100 times higher than that of H_2_O_2_ signaling in inactivating PTP-1B [57, 58]. In addition to being kinetically more efficient, O_2_^•−^ is chemically more specific than H_2_O_2_ in this process as the catalytic site of PTP-1B is surrounded by positively charged residues [59]. This provides an efficient fine-tuning ability of O_2_^•−^ in regulating signal transduction. PTP1B is known to play a distinct and non-redundant role as a negative regulator of c-Met [60]. Therefore, re-expression of EcSOD, by scavenging O_2_^•−^ and blocking superoxide-mediated PTP1B inactivation, provides an inhibitory regulation of oncogenic signal transduction such as the c-Met pathway.

In comparison to the other two intracellular SODs, EcSOD is the new comer in terms of its tumor suppressive role in cancer and the mechanisms involved are less well understood. The degree of differential expression of this antioxidant in cancer versus normal cells/tissues however, is more pronounced and prevalent than the other SODs in breast cancer. Oncomine gene expression signature analysis identifies EcSOD as one of the top 1% to 6% under-expressed genes in invasive ductal breast carcinomas versus normal tissues from both the TCGA and Curtis datasets (Figure 7). Down-regulation of EcSOD expression in cancer cells has been associated with epigenetic silencing, up-regulation of oncomir microRNA-21, Ras oncogene-mediated gene silencing, chronic estrogen-induced gene suppression, single nucleotide polymorphisms, DNA copy number variation, and loss of heterozygosity as reviewed recently [61]. All of these observations imply that deregulation of EcSOD expression, distribution, or function provides a selective advantage in cancer cells. Furthermore, association of low EcSOD expression levels with poor relapse-free survival as shown in Table 1 underscores the importance of *EcSOD* as a potential tumor suppressor gene, inhibiting the progression of malignant phenotype in human breast cancer. However, the mechanisms of how EcSOD loss could promote oncogenesis is not fully understood.

## CONCLUSIONS

Taken together, our study shows that ROS contributes to HGF-stimulated c-Met activation and that overexpressing the extracellular antioxidant enzyme, EcSOD suppresses this oncogenic cancer-fibroblast interaction. Modulating the redox tumor microenvironment not only inhibits the oncogenic c-Met pathway in cancer cells but also targets the redox-mediated activated phenotype of CAFs. ROS as merely a damaging bystander species that induce cytotoxic effects does not provide a complete picture of their biological effects. Rather, they should be considered as critical players in fine-tuning cellular signaling events in a temporal and purposeful manner in cancer. Future studies to unravel the specific modifications of signaling molecules/factors by O_2_^•−^ and H_2_O_2_ and how EcSOD regulates oncogenic signal transduction pathways will help to identify specific redox-mediators as potential cancer targets.

## MATERIALS AND METHODS

### Cell lines, growth conditions, and reagents

Human mammary epithelial cell line, MDA-MB231 and MDA-MB468 were obtained from the American Type Cell Culture Collection (Manassas, VA, USA). A firefly luciferase expressing stable cell line, MDA-MB231.luc, was previously generated as described [62]. Stable EcSOD overexpressing MDA-MB231 cell line, Ec.20 were generated as previously described [3]. Reduction mammoplasty fibroblasts expressing human HGF (RMF-HGF) and their parental normal fibroblasts (RMF) were generated by Dr Charlotte Kuperwasser (Tufts University, Boston, MA, USA) [24]. MDA-MB231 were maintained in RPMI1640 (or DMEM when co-cultured with fibroblasts) containing 10% FBS and 1X Pen/Strep. MDA-MB468 and RMF and RMF-HGF fibroblasts were grown in DMEM + 10% fetal bovine serum media and 1X Pen/Strep. All cells are maintained at 37°C, 5% O_2_.

Antibodies: anti-total cMet (Cell Signaling cat # 4560), anti-phopho c-Met at Y1235/1235 (Cell Signaling, Cat # s25728). Antibody for EcSOD was a kind gift from Dr. James Crapo from National Jewish Medical and Research Center,Denver, CO, USA. Rabbit polyclonal anti-human b-actin (Sigma-Aldrich, St Louis, MO, USA). Anti-Nox4 monoclonal rabbit antibody [UOTR1B493] from Abcam (#ab133303).

Reagents: Diphenyleneiodonium chloride (DPI) (Sigma #D2926), recombinant human Hepatocyte Growth Factor (EMD Millipore #GF116), MnTE-2-PyP (a kind gift from Dr. James Crapo), N-acetyl cysteine (NAC, MP Bio #02100098).

### 3D culture and co-culture

### Mono-culture

Mammary epithelial cell were propagated in 3D culture using the ‘on-top assay’ as described [63]. Briefly, 4-chamber slides were coated with 150 uL of Matrigel (BD Biosciences, San Jose, CA, USA). Two-dimensional cultured cells were trypsinized and suspended single cells were then overlaid on the Matrigel at 2 × 10^4^ cells per chamber. Culture media was replaces every 2 days as described [3]. Protocol was also adapted to the μ-Slide angiogenesis with ibiTreat (#81506, iBidi USA, Fitchburg, Wisconsin, USA) using 10 uL of Matrigel and overlaying 2.5 × 10^3^ cells per well.

### Co-culture

Fibroblasts were embedded in 200 uL of Matrigel at 4 × 10^4^ cells per chamber of the 4-chamber slide. Once solidified, 2 × 10^4^ breast cancer cells were seeded on top of the matrix in 3D culture media. In co-culture model using conditioned media, breast cancer cells were seeded n Matrigel as described above except that the 3D culture media was replaced by conditioned media harvested from fibroblasts.

### Prolong co-culture of cancer cells with fibroblasts

Fibroblasts (1 × 10^5^) were grown in the bottom of a 6-well plate and 1 × 10^5^ MDA-MB231 cells were seeded on the 0.4 uM polyester membrane of a transwell insert (BD Bioscience). Cells were co-cultured in DMEM + 10% fetal bovine serum media and 1X Pen/Strep. After 48 h of co-culture, these two cell types were trypsinized, counted and seeded in subsequent co-cultures for a total of 4 passages.

### Cell lysate preparation and western blot analysis

Cell lysates preparation and western blot analysis was performed as previously described [4]. Antibody dilutions were as follows: rabbit polyclonal anti-human EcSOD, 1/5000; rabbit anti-human b-actin, 1/2000; anti-phospho c-Met, 1/1000; anti-total c-Met, 1/1000; anti-Nox4, 1/1000; anti-phospho p44/42, 1/1000; anti-total p44/42, 1/5000; goat anti-rabbit IgG-HRP, 1/50000; and goat anti-mouse IgG-HRP, 1/10000. Densitometry analysis of the signal intensity was performed using ImageJ software (NIH).

### RT–PCR

Total RNA was isolated using Quick RNA Miniprep (Zymo Research, Irvine, CA, USA). Followed by reverse transcription using a High Capacity cDNA Archive Kit (Applied Biosystems Inc., Foster City, CA, USA). Real-time PCR was performed using Maxima SYBR Green (Thermo Fisher Scientific #K0221) on a ABI 7000 Fast machine. The fold change was calculated using the 2^(-ddCt) method using 18S as the reference gene. Primer sequences are as follows:

Nox4 (Forward 5′-AAC ACC TCT GCC TGT TCA TC-3,’Reverse 5′-GAT ACT CTG GCC CTT GGT TAT AC-3′); HGF (Forward 5′-ATG TCA GCG TTG GGA TTC TC-3′; Reverse 5′-TCG GAT GTT TGG ATC AGT GG-3′); CYBA (Forward 5′-CGT CCT GCA TCT CCT GCT-3′, Reverse 5′-GTA GAT GCC GCT CGC AAT-3′); 18S (Forward 5′-CCT TGG ATG TGG TAG CCG TTT-3′, Reverse 5′-AAC TTT CGA TGG TAG TCG CCG-3′); SDF-1 (Forward 5′-GAC CCA ACG TCA AGC ATC TC, Reverse 5′-CGG GTC AAT GCA CAC TTG TC); SMA (Forward 5′-GCG TGG CTA TTC CTT CGT TA, Reverse 5′-TCA GGC AAC TCG TAA CTC TTC TC); PDGFRa (Forward 5′-TGC CTG ACA TTG ACC CTG T, Reverse 5′-CCG TCT CAA TGG CAC TCT CT); FAP (Forward 5′-TGT TTC GGT CCT GTC TAT ATG TG, Reverse 5′-CCC ATC CAG TTC TGC TTT CT).

### Generation of catalytically inactive mutant EcSOD

The doxycycline-inducible catalytically inactive mutant EcSOD expressing MDA-MB231 (iMutEcSOD) were generated using the Lenti-X Tet-On 3G Inducible Expression System (Takara Bio USA, Mountain View, CA, USA). Briefly, this is a two-vector system. One vector expresses a doxycycline-sensing transactivator protein, while the other vector expresses the gene-of-interest in a dox-inducible manner. First, the catalytically inactive mutant EcSOD (N180A, R186A) was mutated. The mutant EcSOD was cloned into the pLVX-TRE3G-IRES vector (Takara Bio USA) by In-Fusion Cloning Kit (#638909, Takara Bio USA) after addition of 5′ and 3′ ends homologous to the vector. Lentiviral particles were generated followed by transduction of the transactivator and mutant EcSOD vector into MDA-MB231 cell line. The transduced cells were selected for double positive cells with puromycin (0.6 μg/mL) and G418 (1.2 mg/mL).

### ROS detection by DHE and CellRox

ROS levels were measured using dihydoethidium (DHE) or CellROX Deep Red Reagent (Thermo Fisher Scientific #C10422). Briefly, fibroblasts were suspended and incubated in 10 μM DHE for 40 minutes at 37°C in the dark or 3 μM CellROX Deep Red for 30 minutes at 37°C in the dark. The fluorescence was then read after excitation (405 nm for DHE and 633 nm for CellROX Deep Red) by flow cytometry using a BD LSRII.

### Amplex Red assay

Extracellular H_2_O_2_ production was measured using the Amplex Red Hydrogen Peroxide Assay kit (#A22188, Invitrogen, Carlsbad, CA, USA). RMF and RMF-HGF fibroblasts (1.8 ×10^5^ cells) were plated in a 12 well format for 48 hours. RMF-HGF cells were treated with 10 μM diphenyleneiodonium (DPI), a pan Nox and flavoprotein inhibitor. After washing with PBS, cells were incubated in 1 mL of the Amplex Red reaction mixture consisting of 50 μM Amplex Red reagent and 0.1 U/mL HRP in HBSS +Ca^2+^/Mg^2+^. Every hour, 300 μL (3 × 100 μL) of the reaction mixture was removed from each sample and put into a 96 well plate. The fluorescence product indicative of the amount of H_2_O_2_ produced was measured using a Tecan infinite M200 pro plate reader at excitation of 560 nm and emission of 590 nm.

### Electron paramagnetic resonance spectroscopy (EPR)

Superoxide levels were measured using 1-hydroxy-3-methoxycarbonyl-2,2,5,5-tetramethylpyrrolidine (CMH). CMH (200 μM) was prepared in Krebs-Hepes Buffer (KHB) with deferoxamine (DF) and diethyldithiocarbamic acid sodium salt (DETC). After washing with KHB +DF/DETC, cells were incubated in CMH for 30 minutes at 37°C. Cells were scraped and resuspended before measuring the EPR signal. The EPR signal was measured using a Bruker E-Scan Table Top EPR spectrometer.

### Glutathione assay

Total and oxidized glutathione (GSH) levels were measured using the GSH/GSSG-Glo kit (#V6611, Promega, Madison, WI, USA) following the manufacturer's protocol. In brief, this assay produces luminescence by coupling glutathione S-transferase with luciferase to produce light in a GSH-dependent manner. Cells were plated and lysed in 96 well white plate to measure total GSH levels (10,000 cells/well) or oxidized glutathione (20,000 cells/well).

### RTK array

Cell lysates were quantified by Bradford assay (Protein Assay, Bio-Rad, USA) and incubated with the PathScan RTK signaling kit array chip (#7949, Cell Signaling Technology, USA) according to the protocol provided by the manufacturer. Briefly, after incubation of the samples, biotin-labeled anti-pan-phospho-tyrosine antibodies and specific anti-phospho-residue antibodies were used to detect phosphorylated proteins captured on each spot on the nitrocellulose membrane. After incubation with DyLight 680-linked streptavidin, the chip was washed, fully dried and imaged using a Li-COR Odyssey imager. The pixel density of the background was subtracted from the pixel density of each spot, and the average of duplicate spots was determined. Next, Signal intensity was calculated by the normalization of mean pixel density in each spot against the pixel density of the positive control.

### Collagen contraction assay

Human mammary fibroblasts were suspended (500,000 cells/mL) in a rat tail collagen I solution (2.2 mg/mL; BD Biosciences, San Jose, CA, USA) as described [64]. MEM (Invitrogen, Carlsbad, CA, USA) supplemented with 1.8 mg/mL NaHCO_3_ (Sigma-Aldrich, St. Louis, MO, USA) and 2.3 mg/mL L-glutamine (Invitrogen) was used as a diluent and pH was adjusted with 0.22 M NaOH. For each replicate, 500 μL cell-collagen mixture was dispensed into a single well of a 24-well plate and incubated at 37°C for 1 hour to facilitate collagen polymerization. Next, the collagen gels were detached from the well using a pipet tip. Culture media (500 μL) was then added and plates were incubated at 37°C in a humidified, 5% CO_2_–containing atmosphere. Images of the collagen matrix were taken at the end of contraction time point, and surface area was measured with ImageJ software (NIH).

### Adenovirus transduction

Overexpression of EcSOD and Nox4 was achieved by adenovirus infection, as previously described with a multiplicity of infection (MOI) of 50 for 48 h in MDA-MB231 cells [4]. Overexpression in MDA-MB468 was achieved using a MOI of 5. Both AdEcSOD and AdNox4 were purchased from University of Iowa Vector Core Facility.

### siRNA transfection

SiRNA transfection was performed using 10 nM siRNA with the Lipofectamine RNAiMAX Reagent (Thermo Fisher Scientific #13778030) following the manufacturers protocol. The siNC#1 (Thermo Fisher Scientific #4390843) and the siNox4 (Thermo Fisher Scientific #4392420; ID#s224159) were purchased from (Thermo Fisher Scientific, Waltham, MA, USA).

### Invasion assay

The *in vitro* invasive properties of the breast cancer cells were performed using the BD Bio-Coat Matrigel invasion assay system (BD Biosciences) as previously described [4]. Briefly, 2 × 10^5^ cancer cells in serum free media were seeded into the Matrigel inserts consisting with 8-um filter pores. After 16 h of incubation, the upper surface of the transwell chambers was removed with a cotton swab and cells that have invaded through the Matrigel were lysed with the Luc-Screen Extended-Glow Luciferase Reporter Gene Assay System kit (Thermo Fisher Scientific, # T1033) and analyzed for luciferase activity using the Tecan Infinite M200 Pro plate reader. In some experiments, invaded cells were counted after being fixed and stained using a crystal violet solution (2% crystal violet, 50% methanol, 10% acetic acid).

### *In vivo* tumor study

Eight-week-old female athymic nude mice were obtained from Harlan Laboratories Inc. (Indianapolis, IN, USA). The nude mice protocol was reviewed and approved by the Animal Care and Use Committee of the University of Iowa. Breast cancer cells (MDA-MB231.luc or Ec.20 at 1 × 10^6^) were injected alone or co-injected with 3 × 10^5^ mammary fibroblasts (RMF or RMF-HGF) into both sides of the 4th mammary fad pads, in the presence of 50% Matrigel. Tumor growth was monitored weekly by bioluminescence imaging as previously described [3].

### Statistical analysis

Statistical analyses were performed using SYSTAT. For some experiments, a single-factor ANOVA followed by post-hoc Tukey test was used to determine statistical differences between means. Statistical analyses were assessed using a two-tailed Student's t test. Results shown are representative of at least three separate experiments each performed in triplicate. Statistical significance was achieved when *P* < 0.05.

## Abbreviations

ROS: reactive oxygen species
O_2_^•−^: superoxide
H_2_O_2_: hydrogen peroxide
^•^OH: hydroxyl radical
EcSOD: extracellular superoxide dismutase
SOD3: EcSOD gene
HGF: hepatocyte growth factor
RMF: reduction mammoplasty isolated normal fibroblasts
RMF-HGF: HGF-overexpressing mammary fibroblasts
RTK: receptor tyrosine kinases
TSPs: thrombospondins
MnTE-2-PyP: Manganese (III) Meso-Tetrakis-(N-Methylpyridinium-2-yl) porphyrin
Nox4: NADPH Oxidase 4
CAFs: cancer-associated fibroblasts
